# State of New-York vs. William Frank

**Published:** 1847-02

**Authors:** 


*State of New-York vs. William Frank.*—In a former number of this Journal we noticed the finding of an indictment, by the grand jury of this county, against a person by the name of Frank, for alledged mal-practice in a case of midwifery. The trial came on at the court of Oyer and Terminer for January, 1847, and resulted in conviction of the defendant of mal-practice, for which he was sentenced to pay a fine of $100, and to be imprisoned in the county jail for ninety days.

We have not time, nor the materials at hand for a full report of the trial; the circumstances, however, as gathered from partial attendance during its progress, and a few notes which have been placed at our disposal, were briefly as follows: The so-called Dr. Frank had been an irregular German Practitioner in this city for the last two years, professing to have a diploma from some German University, but never having been received into fellowship with the profession here. In September last he was called to attend upon a

German woman in labour. A midwife had previously been in attendance, and effected delivery of the body of the child, it being a footling presentation. Dr. Frank proceeded to make violent efforts to accomplish the delivery by manual force, which he continued until the trunk was torn away; he then attempted to deliver the head with the forceps, afterward resuming his efforts with the hands; finally failing in each attempt he left the patient to procure counsel, and death ensued shortly after his departure.

Some days after the burial, the history of the case transpiring, a Coroner's inquest was held; the body was exhumed, and an antopsical examination made by Drs. Pratt and Winne of this city. These gentlemen testified on the trial, that they found in the coffin with the body of the mother, the headless trunk of an infant, with one shoulder dislocated, and the right carpal bones separated from their attachments to the forearm. The entire womb, bladder, and portions of the intestines were extruded from the vagina, An extensive rupture of the vaginal attachment to the uterus existed, and the head of the child was found in the abdomen, the lower jaw gone. No placenta was to be discovered. The dimensions of the pelvic cavity appeared not to be abnormal, and the size of the child's head was less than the average.

It is needless to say that these truly horrible details involve malpractice of an atrocious character; and the defendant may think himself fortunate in escaping with so slight punishment. The indictment charged him with manslaughter in the first and fourth degrees, as well as with mal-practice. The jury, however, brought in a verdict of mal-practice only. That the death of the mother was occasioned by barbarous efforts to effect a forcible delivery, can hardly be doubted. It appeared, in evidence, that she had previously borne children, that she was healthy and comfortable up to the period of labour, and that no deformity of the pelvis existed, the head being under size. By judicious manual aid, by the forceps, or at all events, by embryotomy, under these circumstances, she should have been safely delivered. The tearing away of the body of the child, the dislocation &c., showed the great force that must have employed; and after the vagina had been ruptured, and the head forced by the contractions of the uterus into the abdomen, the uretus itself, with the bladder, must have been brutally drawn out! A suspicion may have been entertained, that a portion of the mischief was done by the midwife, before the attendance of Frank; otherwise, we can scarcely account for his escaping a verdict of manslaughter.

